# How to Approach Secondary Breast Reduction: International Trends and a Systematic Review of the Literature

**DOI:** 10.1007/s00266-021-02243-1

**Published:** 2021-04-05

**Authors:** P. Niclas Broer, Nicholas Moellhoff, Thiha Aung, Antonio J. Forte, Charlotte Topka, Dirk F. Richter, Martin Colombo, Sammy Sinno, Andreas Kehrer, Florian Zeman, Rodney J. Rohrich, Lukas Prantl, Paul I. Heidekrueger

**Affiliations:** 1 Department of Plastic, Reconstructive, Hand and Burn Surgery, Muenchen Klinik Bogenhausen, Technical University Academic Teaching Hospital, Munich, Germany; 2 Division of Hand, Plastic and Aesthetic Surgery, University Hospital, LMU Munich, Munich, Germany; 3 Centre of Plastic, Aesthetic, Hand and Reconstructive Surgery, University of Regensburg, Franz-Josef-Strauß-Allee 11, 93053 Regensburg, Germany; 4 Division of Plastic Surgery, Mayo Clinic, Jacksonville, FL USA; 5 Department of Plastic and Reconstructive Surgery, Dreifaltigkeitskrankenhaus, Wesseling, Germany; 6 Private practice in Buenos Aires, Buenos Aires, Argentina; 7 TLKM Plastic Surgery, Chicago, IL USA; 8 Center for Clinical Studies, University Medical Center Regensburg, Regensburg, Germany; 9 Dallas Plastic Surgery Institute, Dallas, TX USA

**Keywords:** Secondary mammoplasty, Breast re-reduction, Repeat reduction mammoplasty, Breast reduction, Mastopexy

## Abstract

**Background:**

Secondary breast reduction is complex and poses significant challenges to surgeons. Complication rates exceed those of primary reduction, commonly caused by impaired vascular supply of the nipple-areolar complex (NAC). Literature on the topic is scare and provides contradicting recommendations, especially with regard to pedicle choice in cases with unknown primary reduction technique. Aim of this study was to investigate international trends and to compare findings with literature.

**Methods:**

A large-scale web-based questionnaire on international trends in mammaplasty (mastopexy and breast reduction) was designed and distributed to over five thousand surgeons in eight geographic regions. The presented manuscript evaluated information regarding pedicle choice in secondary breast reduction and compared data to literature identified in a systematic review.

**Results:**

The survey was completed by 1431 participants. Overall, secondary procedures were performed in less than 5% or in 5 to 10% of cases. The preferred pedicle for secondary reductions differed significantly between geographic regions (*p*<0.001). The majority of respondents reported to use a superior or supero-medial pedicle (34.8% and 32.2%, respectively). Residual analysis revealed a strong association between the use of an inferior pedicle and procedures performed in North America.

**Conclusions:**

Secondary breast reduction is challenging and there remains international disparity with regard to pedicle choice for secondary procedures. Studies investigating outcome when the primary pedicle is unknown are scarce and provide incoherent recommendations. High-quality data is needed to provide evidence-based practice guidelines.

**Level of Evidence III:**

This journal requires that authors assign a level of evidence to each article. For a full description of these Evidence-Based Medicine ratings, please refer to the Table of Contents or the online Instructions to Authors www.springer.com/00266

## Introduction

Breast reduction mammaplasty is a popular procedure in plastic surgery, with numbers reaching over 500.000 reductions in 2018 worldwide [1]. Primary breast reduction is considered safe and the level of patient satisfaction is high [2]. However, there are several indications for secondary breast reduction, which include asymmetry, poor shape, excessive scarring or a recurrent increase in breast volume after primary reduction procedures [3].

Breast re-reduction is complex and poses significant challenges for surgeons. Complication rates significantly exceed those of primary reduction [4], with necrosis or loss of the nipple areola complex (NAC) being respected by many surgeons. Most of these complications can be attributed to impaired vascular supply of the NAC after revision surgery. Hence, an in-depth understanding of the breasts vascular supply is deemed necessary for the success of the procedure. After primary reduction mammaplasty, the blood supply consists of the original pedicle as well as neovascularization from the surrounding tissue which needs to be taken into account when performing secondary breast reduction [5–7].

Over the past decade, plastic surgeons have suggested how to approach secondary breast reduction; however overall literature on this topic is scarce. Mainly, authors developed guidelines, algorithms and treatment plans based on preferred operative techniques, expert opinions, case reports or on limited case series (Level 4 evidence) [3, 4, 8–14]. Consequently, there is a great disparity in terms of recommendations and there remains debate whether the primary pedicle should be recreated, if using a different pedicle for secondary reconstruction is safe or if relying on random pattern blood supply is feasible.

In order to shed further light onto this controversial topic, the presented study investigates international trends with regard to pedicles used for secondary reconstructions and compares findings with recent literature on this topic.

## Materials & Methods

### Online-Survey

#### Design of Questionnaire

A large-scale online survey was designed to evaluate international trends in mammaplasty (mastopexy and breast reduction) and sent to more than five thousand surgeons, practicing plastic surgery across 77 different countries and eight geographical regions (North America, Latin America, Europe, Africa, Middle East, Central Asia, South East Asia, Oceania). In order to generate comparable data, the survey was designed in accordance to a version published by Rohrich et al. in 2002 [15]. Overall, the survey addressed surgeon demographics (practice region and location, years in practice, type and nature of practice, number of mastopexy/ breast reduction procedures performed annually), international patterns of patient care, surgical technique (primary and secondary mammaplasty), as well as standard perioperative safety measures.

#### Data Collection and Analysis

Survey distribution was performed using an e-mail commercial service provider (Mailchimp, Atlanta, GA, USA). National and international plastic surgical societies were contacted and provided contact information (e-mail) of registered member surgeons or directly forwarded the survey to their members. Data were collected anonymously. This study was conducted in accordance with the Declaration of Helsinki. The survey was conducted over a period of 5 months, from February 1st, 2018, to June 30th, 2018. Reminders for completion were sent out after four and eight weeks. A multitude of data was collected by distribution of this survey. Results of the mastopexy and primary breast reduction section will be presented separately. The presented manuscript evaluates data regarding secondary breast reduction only. Categorical variables were compared by using the Chi-Square-Test of Independence. A *p* value<0.05 was considered statistically significant.

### Literature Review

#### Search Strategy

Pubmed and the Cochrane Library were reviewed for publications on secondary breast reduction (1990-2020). The following search strategy was conducted: ((((((secondary breast reduction[Title/Abstract]) OR (breast re-reduction[Title/Abstract])) OR (repeated breast reduction[Title/Abstract])) OR (secondary reduction mammaplasty[Title/Abstract])) OR (recurrent mammary hyperplasia[Title/Abstract])) OR (rereduction mammaplasty [Title/Abstract])) OR (repeat reduction mammaplasty[Title/Abstract])) OR (repeated bilateral reduction mammaplasty[Title/Abstract]). Search results were filtered with respect to title, abstract, and content. All articles were then reviewed for patient characteristics, surgical technique, and outcome. Due to the scarcity of literature, all studies with prior breast reduction and subsequent removal of additional tissue were included. Studies with a focus on breast reduction in adolescents or juveniles of 18 years of age and younger were excluded. Results were limited to original articles published in English language. Comments or panel discussions were excluded from analysis. Two authors (N.M., P.H.) independently screened the titles and the abstracts for eligibility which were identified in the electronic database search. The collected article reference lists were used to identify additional studies. The last search date was January 23, 2021.

#### Data Extraction

The data extracted included author, date, study title, study type, study regional location, sample size, demographic data, years since primary reduction, resection weight, procedure (pedicle) performed, outcome, follow-up length and finally recommendations provided.

## Results

### *Questionnaire: Demographic Data (Tables *1*,*2*,*3*, and *4*)*

**Table 1:** Overview of respondents’ years in practice across geographical regions.

Years in practice	Total (% of respondents, *n*=1431)	North America (% of respondents, n=221)	Latin America (% of respondents, n=430)	Europe (% of respondents, n=502)	Africa (% of respondents, n=39)	Middle East (% of respondents, n=97)	Central Asia (% of respondents, n=74)	South East Asia (% of respondents, n=32)	Oceania (% of respondents, n=36)
0–5	5.5	0	6.0	6.2	10.3	10.3	8.1	0	5.6
6–10	10.0	2.7	14.0	9.0	10.3	18.6	10.8	6.3	0
11–15	15.8	16.3	16.3	14.3	20.5	18.6	18.9	12.5	11.1
16–20	18.0	10.0	18.4	22.5	15.4	12.4	21.6	15.6	11.1
21–25	17.1	25.3	12.1	14.9	17.9	21.6	9.5	28.1	50.0
> 25	33.6	45.7	33.3	33.1	25.6	18.6	31.1	37.5	22.2

**Table 2:** Overview of respondents’ type of practice across geographical regions.

Type of practice	Total (% of respondents, n=1431)	North America (% of respondents, n=221)	Latin America (% of respondents, n=430)	Europe (% of respondents, n=502)	Africa (% of respondents, n=39)	Middle East (% of respondents, n=97)	Central Asia (% of respondents, n=74)	South East Asia (% of respondents, n=32)	Oceania (% of respondents, n=36)
Large plastic surgery practice (≥6 surgeons)	8.1	2.7	7.0	11.2	5.1	7.2	12.2	12.5	5.6
Small plastic surgery group (2–5 surgeons)	24.7	17.2	26.5	31.5	10.3	16.5	12.2	31.3	11.1
Solo	43.0	58.8	44.2	31.5	64.1	48.5	44.6	43.8	50.0
Solo practice–shared facility	15.9	13.6	16.7	13.9	15.4	18.6	23.0	6.3	33.3
Other (e.g., multispecialty group, academic, military):~multispeciality	8.4	7.7	5.6	12.0	5.1	9.3	8.1	6.3	0

**Table 3:** Overview of the relative amount of cosmetic and reconstructive procedures performed by the respondents across geographical regions.

Nature of practice	Total (% of respondents, n=1431)	North America (% of respondents, n=221)	Latin America (% of respondents, n=430)	Europe (% of respondents, n=502)	Africa (% of respondents, n=39)	Middle East (% of respondents, n=97)	Central Asia (% of respondents, n=74)	South East Asia (% of respondents, n=32)	Oceania (% of respondents, n=36)
100% cosmetic	25.4	29.0	22.3	25.9	5.1	40.2	29.7	18.8	11.1
100% reconstructive	2.0	2.7	1.9	2.4	0	0	0	0	5.6
25% cosmetic, 75% reconstructive	15.0	12.7	9.3	22.1	35.9	1.0	14.9	25.0	5.6
50% cosmetic, 50% reconstructive	19.0	22.6	18.1	16.5	33.3	17.5	16.2	3.1	50.0
75% cosmetic, 25% reconstructive	38.6	33.0	48.4	33.1	25.6	41.2	39.2	53.1	27.8

**Table 4:** Annual number of primary mammaplasties (mastopexy/ breast reduction) performed by respondents on an annual basis across geographical regions.

Annual number of mastopexy/ breast reduction	Total (% of respondents, n=1431)	North America (% of respondents, n=221)	Latin America (% of respondents, n=430)	Europe (% of respondents, n=502)	Africa (% of respondents, n=39)	Middle East (% of respondents, n=97)	Central Asia (% of respondents, n=74)	South East Asia (% of respondents, n=32)	Oceania (% of respondents, n=36)
1–50	64.3	52.9	67.7	65.7	79.5	55.7	73.0	84.4	44.4
51–150	31.0	39.8	28.1	31.1	15.4	42.3	21.6	3.1	38.9
151–250	3.6	4.5	2.8	2.4	5.1	2.1	5.4	12.5	16.7
251–350	0.8	1.8	1.4	0.4	0	0	0	0	0
> 350	0.3	0.9	0	0.4	0	0	0	0	0

One thousand four hundred and thirty-one surveys were fully completed and returned, corresponding to a response rate of 29%. Responses from individual countries were grouped into eight geographical regions, resulting in *n* = 221 (15.4%) responses from North America, *n* = 430 (30.0%) responses from Latin America, *n* = 502 (35.1%) responses from Europe, *n* = 39 (2.7%) responses from Africa, *n*=97 (6.8%) responses from the Middle East, *n*=74 (5.2%) responses from Central Asia, *n*=32 (2.2%) responses from South East Asia, and *n*= 36 (2.5%) responses from Oceania. Close to 100% of participants were plastic surgeons (Specialty of respondents: Plastic Surgery 99.1%; General Surgery 0.6%; Gynecology 0.1%; Maxillo Facial Surgery 0.1%; other 0.1%).

Over 50% of all responding surgeons had more than 20 years of experience (Table 1). The majority of respondents worked in a private practice (Table 2). Most respondents worked in an aesthetic, rather than a reconstructive setting. Most participants performed ≥ 75% of all annual procedures as aesthetic procedures (Table 3). The annual number of primary mammaplasties (mastopexy/ breast reduction) performed by respondents varied across the geographical regions (Table 4). Most participants (64.3%) performed < 50 procedures per year, while 31% of all surgeons performed between 51 and 150 procedures annually.

### *Questionnaire: Secondary Breast Reduction *(Figs. 1, 2, 3)

**Fig. 1:**
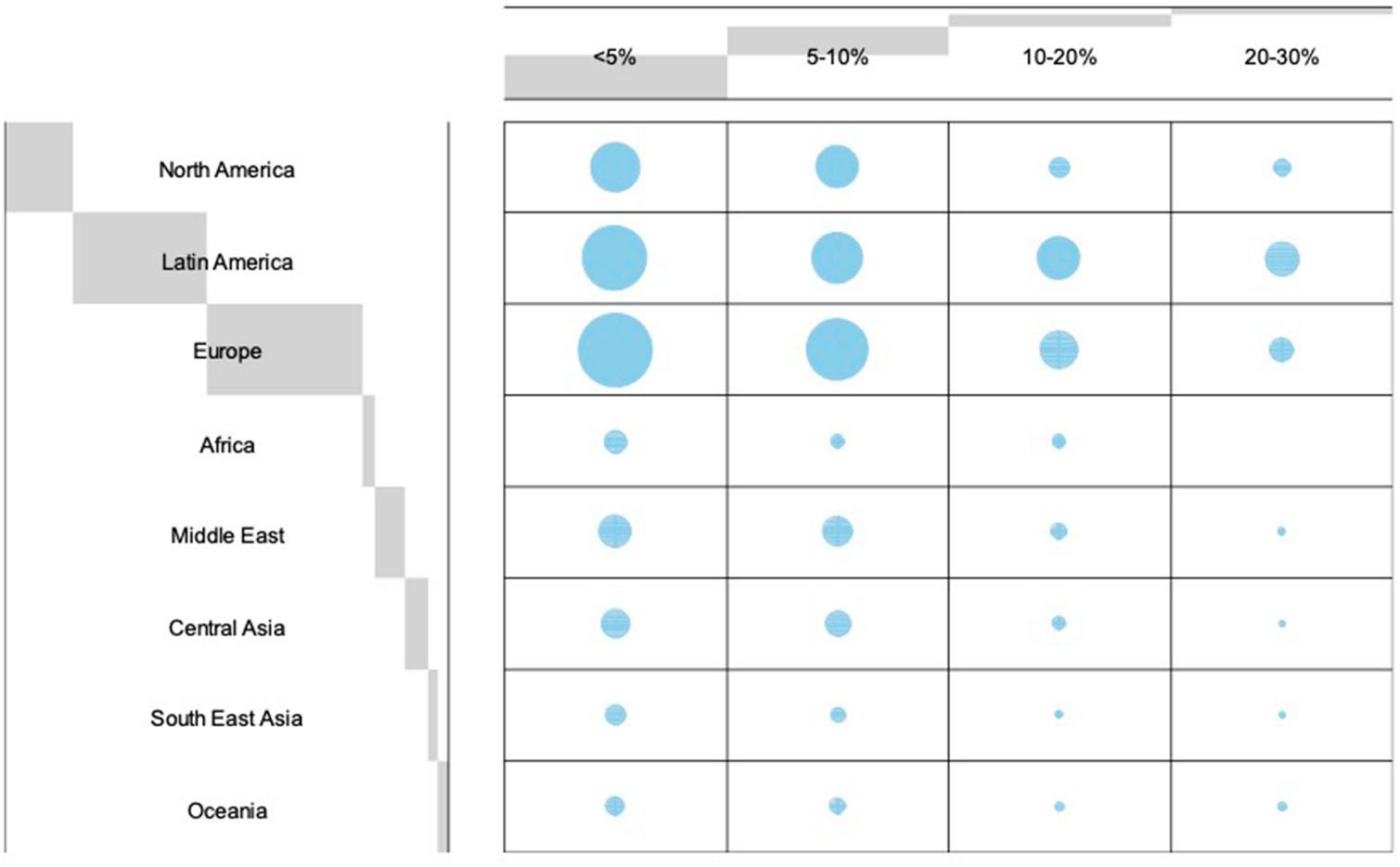
Balloon Plot depicting regional differences in the number of secondary mammaplasties performed, relative to the annual number of primary mastopexy/ breast reduction procedures. The distribution varied significantly across the geographical regions investigated (*p*<0.001)

**Fig. 2:**
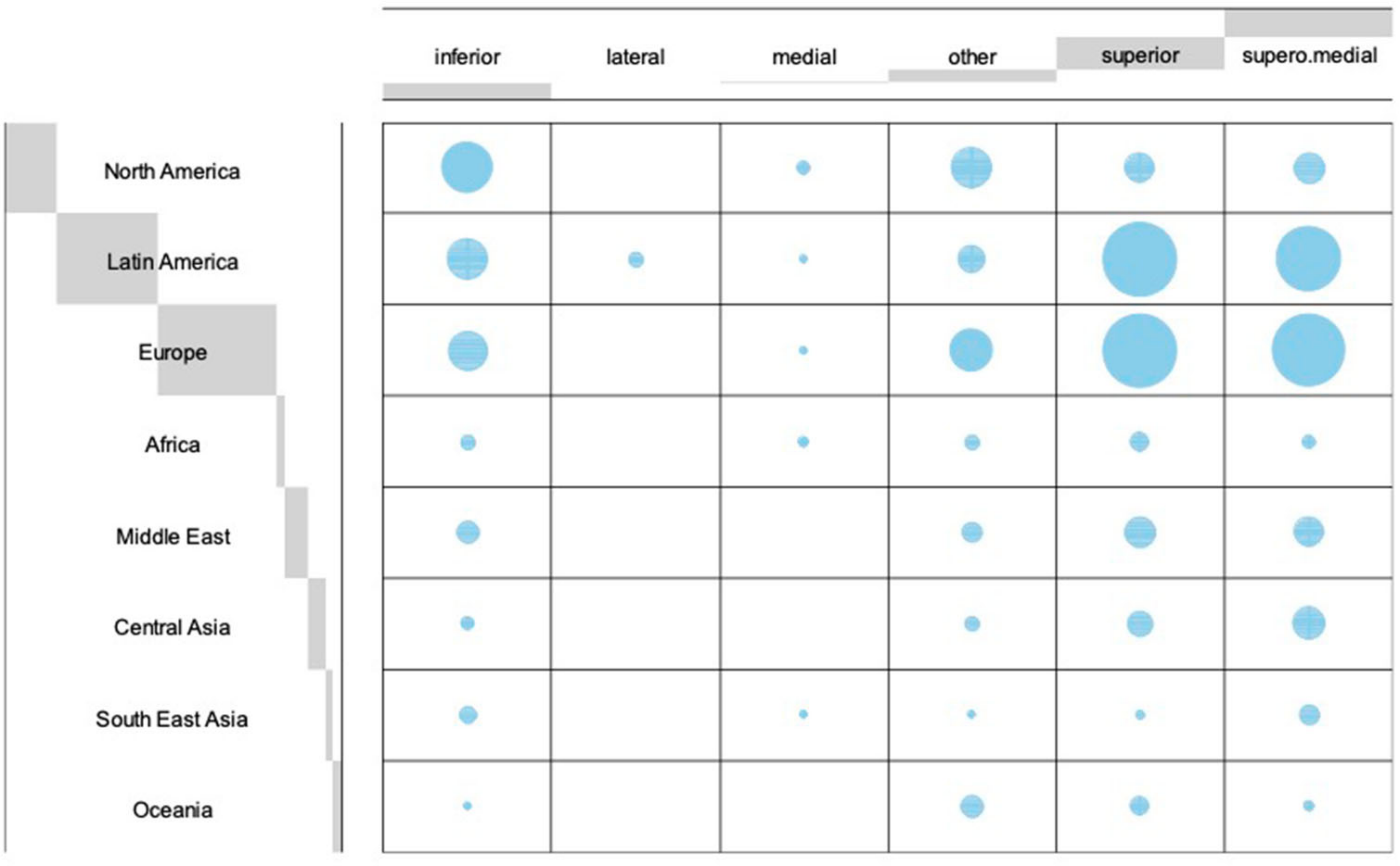
Balloon Plot depicting regional differences in the choice of pedicle for secondary breast reduction if previous surgical technique was unknown. The preferred pedicle for secondary reductions differed significantly between regions (*p*<0.001)

**Fig. 3:**
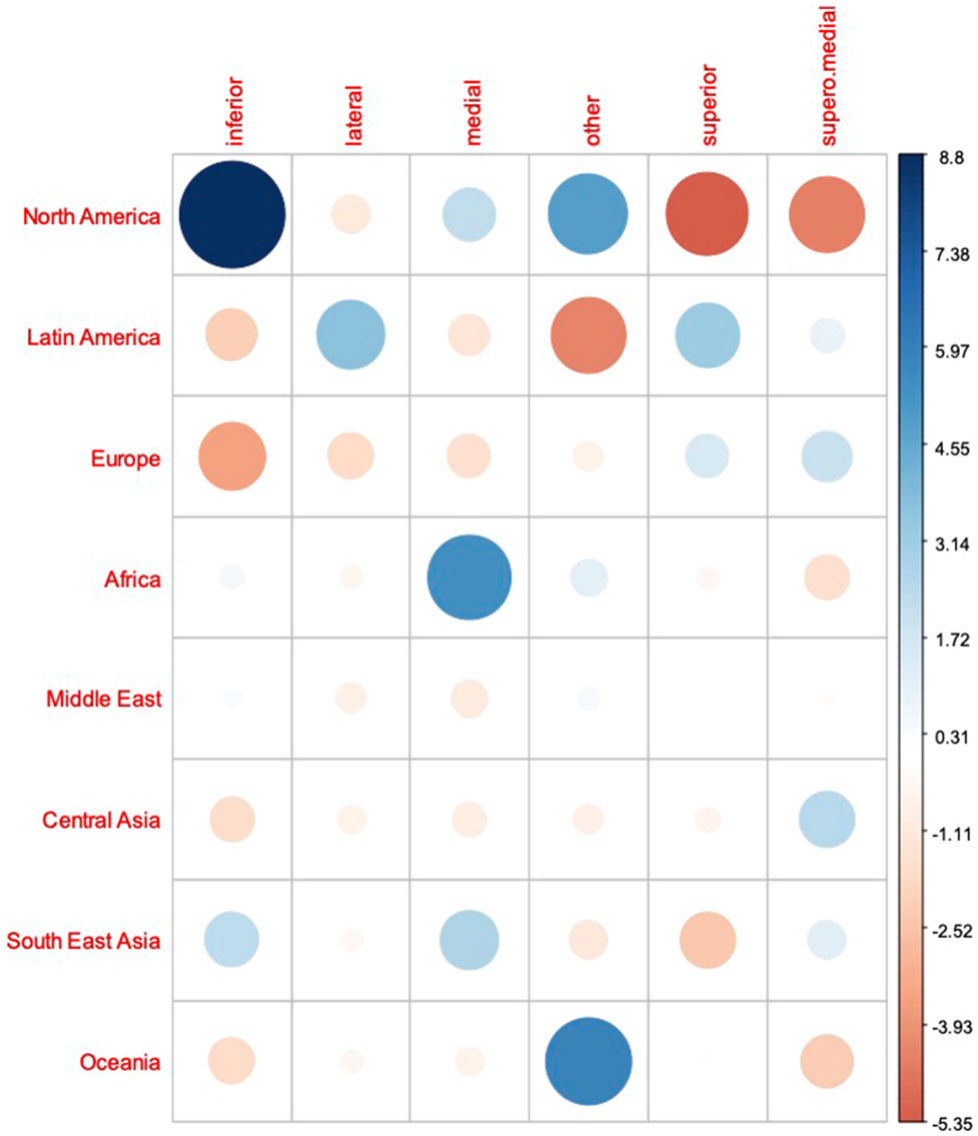
Graphical residual analysis of pedicle choice depending on geographic region. Positive association between categories is indicated by blue color, negative association by red color. The size of the circle and transparency of the color refer to the strength of the association between the categories

Worldwide, the majority of respondents reported that they performed secondary mammaplasty in less than 5% or in five to ten % of their cases (Fig. 1). The distribution varied significantly across the geographical regions investigated (*p*<0.001). The preferred pedicle for secondary reductions differed significantly between regions (*p*<0.001). For cases in which the primary pedicle was unknown, overall the majority of respondents reported to use a superior or supero-medial pedicle (34.8 and 32.2%, respectively) in secondary reduction procedures. In fact, all regions preferred either of these two techniques, except for respondents from North America and Oceania. Surgeons from North America most commonly used the inferior pedicle (42%), whereas respondents from Oceania performed secondary breast reduction using other, not further specified techniques (50%) (Fig. 2). Residual analysis revealed strong association between the use of an inferior pedicle only with North America (Fig. 3).

### *Literature Review (Fig. **4**, Table **5**)*

**Fig. 4:**
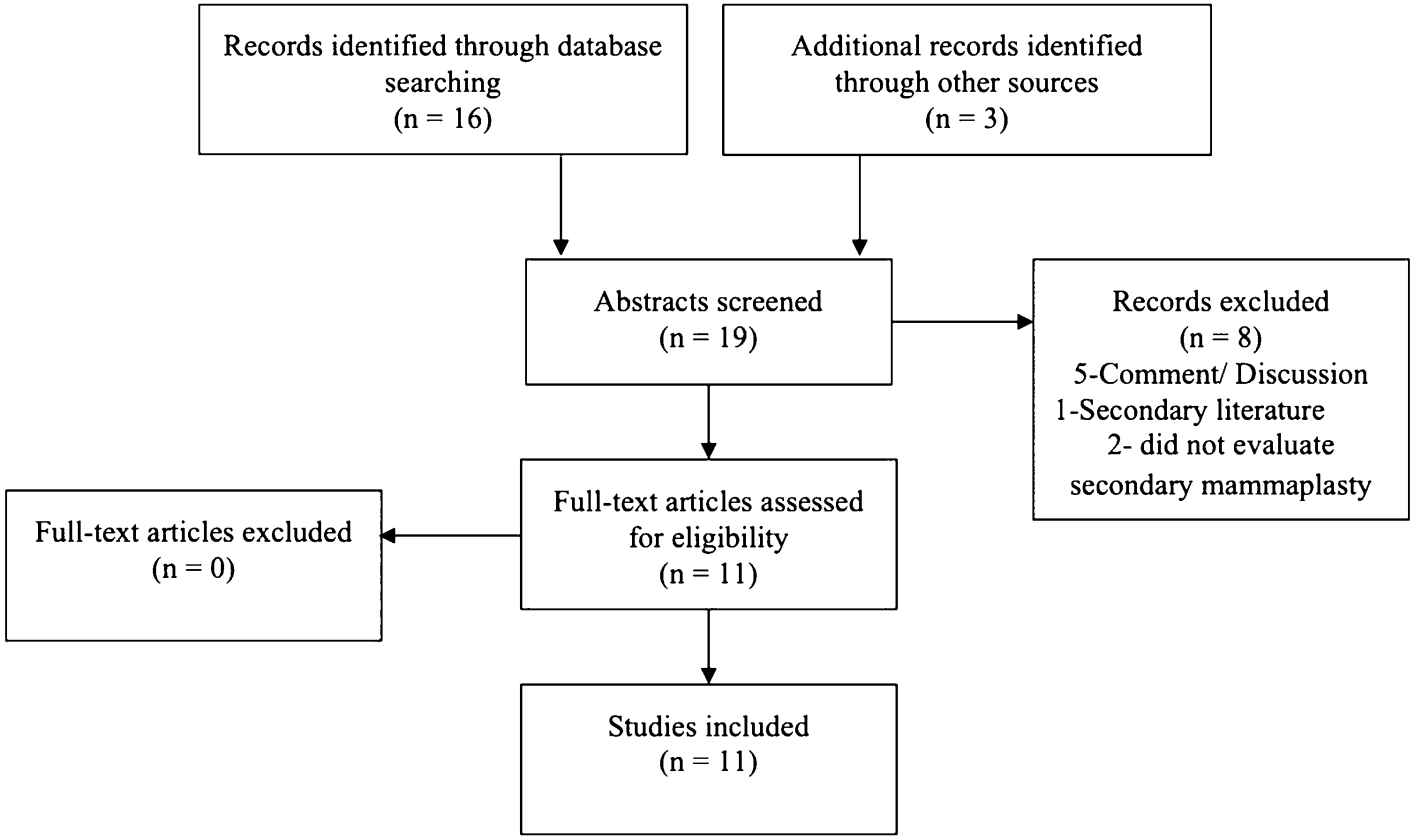
Detailed citation attrition diagram depicting the search strategy

**Table 5:** Overview of studies on secondary breast reduction and detailed study information

Author [Ref]	Year	Location	Design	Patients (n)	Age (y)	Resection weight* (g)	First reduction (y previous)	FU (m)	Pedicle/ Procedure	Complications	Recommendation
Lejour [8]	1997	Belgium	Case-series	10	42	289	8	–	UP n=10 all Patients treated with vertical mammaplasty and superior pedicle	–	Vertical mammaplasty with superior pedicle is safe in secondary reduction; Liposuction is not contraindicated
Hudson [9]	1999	South Africa	Case-series, retrospective analysis	16	29	325	3	>60	IWE n=8 SP n=5 OP n=2 UP n=1	MC n=3 NAC loss n=1, MC n=2 NAC loss n=1, congested NAC n=1 –	For NAC repositioning use SP; If unknown consider FNG; If NAC repositioning is not necessary, perform IWE
Losee [10]	2000	USA	Case-series, retrospective review	10	47	458	15	60	SP n=3 OP n=7	MC n=1 MC n=3	Secondary reduction mammaplasty is safe with either similar or different technique.
Rohrich [11]	2003	USA	Case-report, CME article	2	42	–	16	6, 9	Liposuction and IWE n=2	–	Resection < 500g or Pseudoptosis: IWE; Resection >500g and NAC repositioning: SP if known, otherwise FNG
Patel [4]	2010	USA	Case series, Viewpoint	8	49	695	16	–	SP n=3 OP n=5	NAC necrosis n=1, major seroma/ abscess n=2 –	FNG for repeated bilateral reduction may be the technique of choice
Ahmad [12]	2012 -	Canada	Case series, retrospective review	25	38	332	8	6	SP n=9 OP n=2 UP n=11 all above patients treated with vertical scar reduction mammaplasty and de-epithelialized superior pedicle; IWE n=3	– – – – –	Despite Wise pattern method for the primary breast reduction, vertical techniques for revisions are safe in secondary mammaplasty
Sultan [27]	2013	USA	Case-series	15	47	252	13	–	OP n=4 UP n=11 all patients received vertical mammaplasties with superior and/or superomedial pedicles	MC n=1 –	Pseudoptosis: IWE; NAC repositioning: de-epithelialized superior pedicle + IWE; Liposuction as an adjunct
Mistry [3]	2017 -	New Zealand	Case series, retrospective review	90	45	247	14	6	IWE n=18 SP n=12 OP n=1 IWE +NAC de-epithelialization+ Lipo n=59	– – MC n=1 MC n=1	NAC elevation by de-epithelialization rather than using pedicle; Breast tissue removal using IWE + Liposuction; No skin excision horizontally below inframammary fold
Ghareeb [28]	2017	USA	Case-series, retrospective review	37	–	226	2	–	SP n=26 breasts OP n=5 breasts UP n=4 breasts, IWE n=5 breasts	MC n=4 MC n=1 – –	A conservative superior or central mound pedicle can be used regardless of initial pedicle
Can 13]	2018	Turkey	Case-report	1	48	–	13	3	UP n=1	–	For pseudoptosis IWE; For NAC elevations < 5 cm: superior pedicle and vertical reduction technique; Inverted-T-scar technique is not recommended; however, if required, perform horizontal skin excision superior to current scar
Spaniol [14]	2019	USA	Retrospective cohort analysis	30	40	464	9	5	SP n=15 MCM reduction + SP n=5 IWE n=1 UP n=9 (vertical bipedicle n=1, superomedial n=1, IWE n=1, MCM n=6)	MC n=4 – – MC n=4	If the primary pedicle is unknown, the MCM technique is an excellent option

*FNG* Free nipple graft;* FU* Follow-Up;* IWE* Inferior Wedge Excision* MC* minor complications including delay in wound healing, delay in the return of nipple sensitivity, mild fat necrosis, minor necrosis areolar edge, dog-ear, small hematoma;* MCM* modified central mound (including superior and inferior pedicle);* m* months;* NAC* Nipple areolar complex;* OP* other pedicle as first reduction;* Ref* Reference;* SP* Same pedicle as first reduction;* UP* unknown pedicle;* y* years

^*^per breast

Literature review according to the defined search criteria yielded 19 articles. After applying inclusion and exclusion criteria, 11 articles were included in this investigation. Eight articles were excluded on abstract review. The detailed citation attrition diagram is depicted in Fig. 4.

The manuscripts included evaluated a total of 244 patients requiring secondary breast reduction surgery. Of these articles, one was a retrospective cohort analysis while all others were case-reports, case-series or retrospective reviews of case-series. One manuscript was a continuing medical education (CME) article which also contained two separate case-reports. The average age of patients was 42.7 years. Primary reduction was performed a mean of 10.6 years prior to secondary reduction. On average, in the studies that reported resection weight during secondary mammaplasty, 365 g of breast tissue were excised per breast. Major complications, defined as NAC necrosis/ loss (*n*=3), NAC congestion (*n*=1), as well as major seroma and abscess (each *n*=1) occurred in 6/244 patients, accounting for 2.5 %. One case of NAC loss and one case of NAC congestion were found in re-reductions using a different pedicle as compared to the primary procedure. One case of NAC loss and one case of NAC necrosis occurred in patients undergoing secondary breast reduction using the same pedicle. In all three cases of NAC loss or necrosis, secondary reduction was performed using an inferior pedicle, after either a primary inferior pedicle (*n*=2) or a primary superomedial pedicle (*n*=1). Minor complications, as defined as delay in wound healing, delay in the return of nipple sensitivity, mild fat necrosis, minor necrosis of the areolar edge, dog-ear, or small hematoma were found in 23/244 patients, accounting for 9.4% of all cases. Recommendations as to which pedicle should be utilized in secondary breast reduction procedures differed largely.

## Discussion

The optimal operative technique in secondary breast reduction is a controversially debated topic. There is a scarcity of literature, which is based on inconclusive evidence that provides contradicting recommendations, especially with regard to pedicle choice in cases with unknown primary reduction technique. Of the few studies that focus on re-reduction mammaplasty in adults, most merely provide level 4 evidence. As a result, there exists much inconsistency as to which surgical approach to apply.

Anatomy lays the groundwork for surgery and reasons for the divergence of surgical procedures might be found within anatomical studies. In fact, differing anatomical works have been publicized in this regard. Most anatomic studies conclude that the breast has a dual vascular supply based on an internal thoracic and an intercostal supply medial to the NAC, as well as a lateral thoracic supply including other minor contributors lateral to the NAC [16–18]. However, there is variation with regard to the pedicle recommended even for primary breast reduction surgery based on vascular anatomy. Perfusion of the NAC can be highly variable, with different patterns existing even between two breasts of the same patient. According to the cadaveric study of van Deventer et al., an inferomedial pedicle is the mainstay for breast reduction [17]. On the other hand, the results of O´Dey et al propose benefits of a full-thickness glandular dermal superolaterally based pedicle [18]. More recent studies, however, support that perforators from the internal thoracic system contribute most to the main vascular supply of the NAC and an in vivo study from Seitz et al., based on MRI images, provides compelling evidence that the main vessel supply for the NAC is superomedial [19].

Interestingly, while older studies recommended the use of free nipple grafts if NAC repositioning was required during secondary breast reduction, more recent studies seem to have left this dogma. Of the more recent studies reviewed in this manuscript, most support a superior or superomedial pedicle for secondary breast reduction, especially when repositioning of the NAC is required. In 2015, the study group of Lista et al. [20] reported that they had now performed over 40 secondary breast reductions using their technique of vertical scar reduction mammaplasty that was previously described by Ahmad et al. in 2012 [12], which incorporates a superiorly based dermoglandular pedicle if the NAC needs repositioning <5 cm and a superomedial pedicle if repositioning >5 cm is required, without encountering significant complications to the NAC. This approach was feasible and safe even in cases of repeated breast reduction, where a different skin resection pattern, such as an inverted T scar, and/or a different pedicle was used during the primary breast reduction [20, 21]. Importantly, Austin et al. state that this technique of pedicle selection limits the length-to-base width ratio of the superior and superomedial dermoglandular flap to 1:1 or even less, thus ensuring adequate blood supply to the NAC [21].

Two more recent studies on this topic have recommended the use of different approaches. In 2017, Mistry et al. proposed NAC elevation by de-epithelialization only, therefore maintaining a random pattern blood supply to the NAC, rather than (re-)creating a pedicle, in addition to breast tissue resection using an inferior vertical wedge excision and liposuction if needed [3]. Their approach is based on evidence from studies that reviewed nipple-sparing mastectomy (NSM) in patients who previously had breast reduction or mastopexy [22, 23]. These studies suggest that the NAC can survive even after having previously been circumferentially incised, due to revascularization across the scar tissue [22, 23]. In addition, Spear et al. demonstrated the feasibility of NAC elevation by simple de-epithelialization in this patient population [24]. Intriguingly, Mistry et al. imply that their approach is actually no different from the one previously proposed by the study group of Ahmad et al. and Lista et al. [12, 20] As stated previously, Ahmad et al. recommend creating a superior pedicle when the previous pedicle is unknown. In fact, according to Mistry et al., what the authors describe is de-epithelialization and elevation of the nipple but maintaining the blood supply not on a new “pedicle” but on a random pattern blood supply [3].

In 2019, Spaniol et al. performed the only retrospective cohort analysis comparing outcome of secondary breast reduction when the primary pedicle was known vs. when it was unknown [14]. When the pedicle was known, they included at least the primary pedicle in the operative plan. When unknown, they performed a modified central mound (MCM) reduction technique. According to the authors, this technique respected the blood supply of the NAC by preserving any remaining vascularity that was present within the central mound tissue while also conserving superior and inferior vascular pedicles [14]. They found no difference in outcome between both groups and concluded that their MCM technique is an excellent option for cases with unknown primary pedicle.

The data obtained from the questionnaire introduced in this manuscript show regional disparity with regard to the pedicle used for secondary breast reduction in cases with unknown primary pedicle. Interestingly, while we found that most of the studies included in the literature review were based in the U.S. and recommended the use of a superior- or superomedial pedicle for re-reduction surgeries, conversely, the majority of surgeons from North America stated to use an inferior pedicle for secondary reduction. This suggests that recommendations are either not commonly known or are disregarded due to their low-quality evidence. Previous studies have shown that the Wise pattern with an inferior pedicle technique is the most popular technique for primary reduction in the U.S. [15, 25, 26]. Surgeons are therefore probably most comfortable with this technique and thus perform it also in secondary reduction. Also, for patients that had primary surgery in the U.S., but in cases where the primary pedicle is unknown, chances are highest that creation of an inferior pedicle safely recreates the primary pedicle.

For Latin America, the Middle East, Central Asia and Europe, our data suggests that the superior or superomedial pedicle is the preferred options for secondary breast reduction.

The presented study and literature review highlight the need for quality data on this topic. Ultimately, no clear recommendations can be drawn from the currently available data. Future studies should conduct prospective clinical trials and investigate the different pedicles used in secondary breast reduction also with regard to the amount of breast tissue resected, as well as the time passed since primary reduction surgery. These two factors are likely to significantly impact outcome after secondary reduction surgery; however, to our knowledge no study has adequately addressed these factors, and literature review revealed that studies were not comparable in this regard.

## Conclusion

Secondary breast reduction is challenging and there remains international disparity with regard to pedicle choice for secondary procedures. Studies investigating outcome when the primary pedicle is unknown are scarce, and provide incoherent recommendations. High-quality data is needed to provide evidence-based practice guidelines.
